# Environmental temperature and seasonal variations as risk factors for Achilles tendon rupture: a multi-center retrospective study in China

**DOI:** 10.3389/fpubh.2025.1732352

**Published:** 2026-01-08

**Authors:** Wan Zhou, Zili Chen, Chunyun Zhuo, Jie Liu, Mi Yang, Shunji Gao, Huijuan Xiang, Li Yang, Rui Du

**Affiliations:** 1 Department of Ultrasound, General Hospital of Central Theater Command, Wuhan, Hubei, China; 2 Department of Ultrasound, Gao Mi People's Hospital, Weifang, Shandong, China; 3 Department of Ultrasound, International Mongolian Hospital of Inner Mongolia, Hohhot, Inner Mongolia, China; 4 School of Medicine, Wuhan University of Science and Technology, Wuhan, Hubei, China

**Keywords:** Achilles tendon rupture, environmental temperature, seasonal variation, tendon biomechanics, sports-related injuries

## Abstract

**Objectives:**

Achilles tendon rupture (ATR) is a common sports-related injury influenced by intrinsic and extrinsic factors. Environmental temperature and seasonal variations may play a role in ATR incidence, but their relationship remains underexplored. This study aimed to determine whether ambient temperature and seasonal changes are associated with the incidence of ATR.

**Methods:**

This retrospective study analyzed 379 ATR cases across three provinces in China—Hubei, Shandong, and Inner Mongolia—from October 2011 to June 2024. Temperature data were classified into five groups (−12.9 to −3.3 °C, −3.2 to 6.4 °C, 6.5–16.1 °C and 16.2–25.8 °C, 25.9–35.5 °C), and seasons were categorized into spring, summer, autumn, and winter. ATR incidence was assessed across these categories, with statistical significance evaluated using chi-squared and binomial tests.

**Results:**

ATR incidence was highest at temperatures 16.2–25.8 °C, a pattern that was observed consistently across Shandong and Inner Mongolia Provinces (*P* < 0.001), However, the highest incidence occurs at temperatures between 25.9 and 35.5 °C in Hubei. Conversely, the injury rate is lowest at temperatures ranging from −12.9 to −3.3 °C, a pattern observed in all three provinces of Hubei, Shandong, and Inner Mongolia. Seasonally, spring exhibited the highest incidence (*P* = 0.001), while winter showed the lowest (*P* = 0.002). Regional differences were evident, with peak ATR rates occurring in May (Hubei), June (Shandong), and September (Inner Mongolia). Patients aged 30–39 years or those who are overweight were most affected, with sports-related injuries, particularly basketball, accounting for the majority of cases.

**Conclusions:**

In this retrospective analysis of a three-province Chinese cohort, we observed that environmental temperature and seasonal variations were associated with ATR incidence, with the lowest rates at very low temperatures and higher incidence at mild-to-warm temperatures, particularly during springtime. These findings highlight the importance of validating these observations through larger, population-based studies.

## Background

The Achilles tendon, the largest and strongest tendon in the human body, is renowned for its robust size and tensile strength. However, despite these remarkable characteristics, it remains susceptible to rupture, particularly among individuals engaged in vigorous physical activities (1). The incidence of acute Achilles tendon rupture (ATR) reported in the literature varies widely, ranging from 7 to 37 cases per 100,000 person-years (2, 3). With an increasing number of people participating in recreational and competitive sports, the annual incidence of ATR has shown a steady increase (4, 5). Notably, ATR occurs more frequently in males than females, and its overall prevalence has risen over time (6, 7).

A variety of risk factors have been associated with ATR, including age, type and frequency of physical activity, a history of Achilles tendinopathy, rheumatoid disease, and the use of specific medications (8–11). Histopathological and imaging studies indicate that most spontaneous ATR arise on a background of chronic degenerative tendinopathy, characterized by collagen disorganization, neovascularization and a failed healing response rather than acute inflammation (12, 13). Aging further alters tendon matrix composition and mechanical properties, leading to inferior Achilles tendon stiffness and impaired ankle function, which may help explain the mid-life peak in ATR incidence (14). Systemic conditions such as rheumatoid disease, obesity and exposure to medications including fluoroquinolone antibiotics and corticosteroids can additionally disrupt tendon homeostasis and have been linked to an increased risk of tendon rupture in observational studies (13, 15). Among these intrinsic and extrinsic factors, high load sports participation remains the most prominent immediate precipitant of ATR (10, 16).

Beyond individual-level risk factors, environmental conditions may modulate ATR risk by shaping physical activity behavior. Device-measured studies and scoping reviews show that season and weather, particularly ambient temperature, daylight duration and precipitation, are important determinants of daily physical activity and sedentary time, with warmer and longer days generally associated with higher levels of moderate-to-vigorous activity and winter conditions with reduced activity (17, 18). Environmental temperature has been shown to influence physical activity levels, particularly in sports and recreational contexts (19, 20), potentially affecting the risk of ATR. Nevertheless, despite increasing recognition of the role of environmental factors, there is a paucity of research exploring the relationship between ATR and variations in daily environmental temperature or seasonal patterns.

This study aims to address this gap by integrating detailed temperature data recorded at the time of injury and investigating the association between environmental temperature, seasonal variation, and the incidence of ATR. Our analysis spans three distinct geographic regions in China—Hubei, Shandong, and Inner Mongolia—to provide a comprehensive assessment. We hypothesized that (1) ATR incidence would show clear seasonal variation, with higher rates in spring and lower rates in winter; (2) ATR incidence would be higher at mild-to-warm temperatures and lower at very low temperatures; and (3) these patterns would be broadly observable across regions with different climatic profiles.

## Materials and methods

### Study design and participants

This retrospective cohort study utilized data from patients diagnosed with ATR at the author's hospital between October 2011 and June 2024. The study protocol was approved by the Medical Ethics Committee of author's hospital (ID: [2024]087-01). Ethics committee explicitly waived the informed consent due to the study's retrospective nature. The inclusion criteria were: (1) Surgical records confirming partial or complete ATR. (2) Availability of complete patient data, including age, gender, height, weight, blood pressure, blood glucose levels, smoking status, type of sports, cause of injury, injury location, and the exact date and time of injury. To ensure that only first-episode, acute ATR events were analyzed, the exclusion criteria were: (1) Any documented history prior to the index rupture of chronic Achilles tendon disorders (such as tendinopathy or insertional disease), inflammatory arthritis, gout, or a previous Achilles tendon rupture or surgery on either side. (2) A history of fluoroquinolone use or local corticosteroid injection around the Achilles tendon.

### Study area

**Figure 1:**
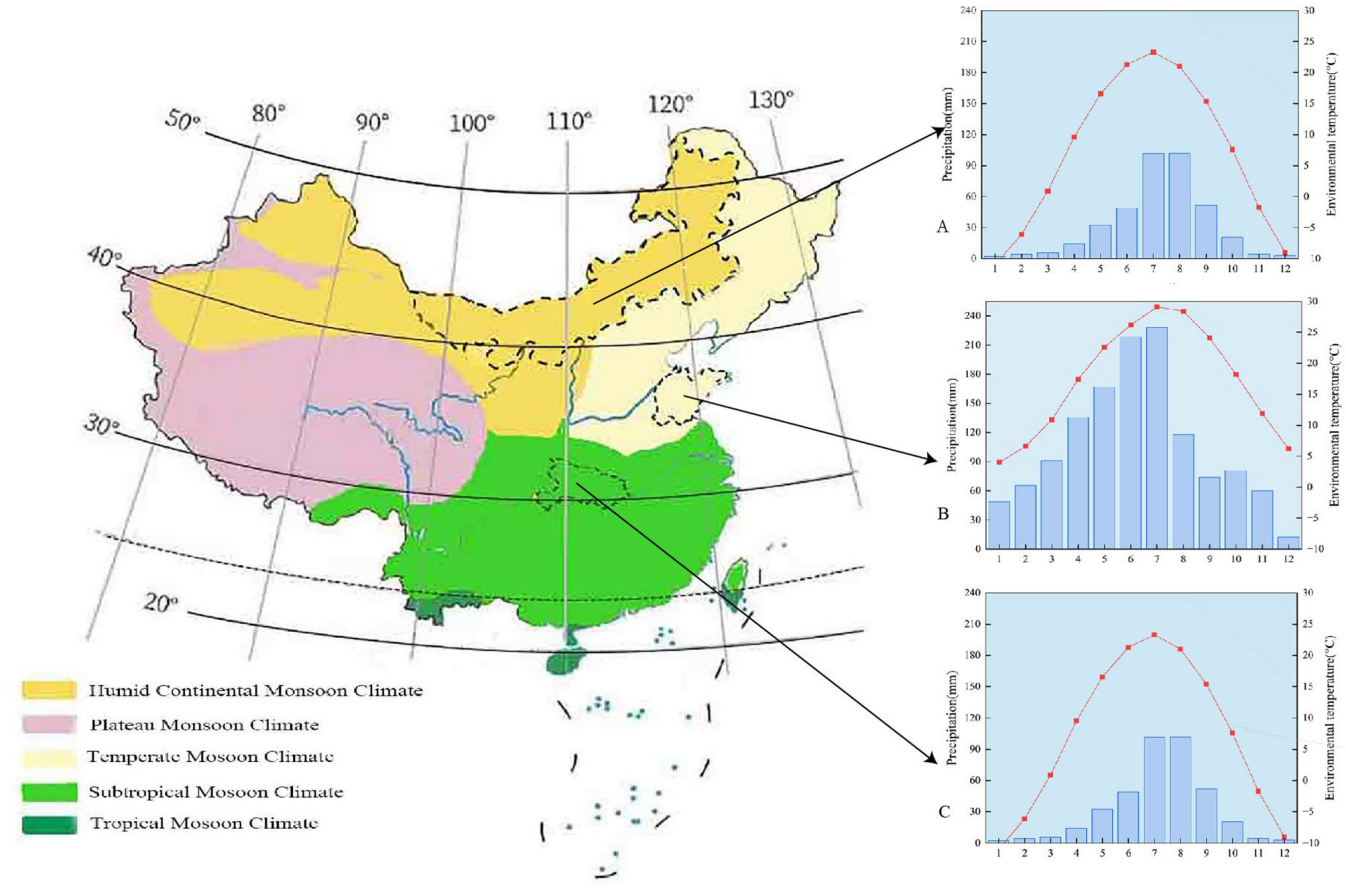
**(A–C)** Distribution of climate types in China, the monthly average temperature and monthly average precipitation in Hubei **(A)**, Shandong **(B)**, and Inner Mongolia **(C)**.

Hubei Province serves as a geographical nexus between eastern and western China, while also bridging northern and southern regions. Situated in the country's central region at 29°05′-33°20′N and 108°21′-116°07′E, Hubei falls within a subtropical monsoon climate zone, characterized by well-defined seasons and a long, hot summer (21). In this study, ATR cases from Hubei were obtained from a tertiary referral hospital in Wuhan, which mainly serves the urban population and surrounding districts. Conversely, Shandong Province, located between the eastern coast of China and the lower reaches of the Yellow River with latitude ranges from 34°22.9′ to 38°24.01′ N and longitude ranges from 114°47.5′ to 122°42.3′ E, experiences a warm temperate monsoon climate with four distinct seasons (22). The Shandong cohort was derived from a large general hospital in Qingdao, representing patients from the city and its nearby urban and suburban areas. In contrast, Inner Mongolia, situated in northern China (37°-53°N, 97.2°-126°E), shares borders with Russia and Mongolia to the north and spans across the regions of Northeast, North, and Northwest China. Geographically delineated by mountain ranges, the region exhibits a predominantly temperate continental monsoon climate characterized by a cold average annual temperature (23). ATR cases from Inner Mongolia were collected at a regional tertiary hospital in Hohhot, which primarily receives patients from the local metropolitan area and surrounding regions. Across all three sites, most participants were local residents or long-term workers living in these areas and were ordinary community-dwelling individuals rather than professional athletes, and they sustained their injuries and underwent surgery at the study hospitals. These varied climatic conditions of hospitals in these three regions provide typical examples for investigating the relationship between ATR and environmental temperature as well as seasonality in China. Regions further south were excluded because they are characterized by relatively stable, year-round warm temperatures with limited seasonal variation, which makes it difficult to assess temperature-related gradients in ATR risk (Figure 1).

### Seasons and temperature assessments

Seasons were categorized based on the method described by Lingfei et al. (24), with spring defined as March to May, summer as June to August, autumn as September to November, and winter as December to February of the following year.

Temperature data were obtained from the European Center for Medium-Range Weather Forecasts (https://www.ecmwf.int/). The environmental temperature at the time of the patient's injury were precisely recorded in accordance with the latitude and longitude of the county where the locality was situated. According to the quintile method temperatures were divided into five categories: Group 1 (−12.9 to −3.3 °C), Group 2 (−3.2 to 6.4 °C), Group 3 (6.5–16.1 °C), Group 4 (16.2–25.8 °C) and Group 5 (25.9–35.5 °C).

### Covariate variables

Several covariates were included in the study: age, gender (female or male), body mass index (BMI), blood pressure, blood glucose, smoking status, and types of sports. Body mass index (BMI; kg/m^2^) was used to classify weight, with participants categorized as normal (< 25.0), overweight (25.0–29.9), or obese (≥30.0). Smoking status was categorized as never smoker or current smoker.

### Statistical analysis

Data analysis was performed using SPSS 27.0 (IBM Corp, Armonk, US). Categorical variables were expressed as numbers (*n*) or proportions (%), with 95% confidence interval (CI), while continuous variables were presented as means ± standard deviations (SD) for normally distributed data. Non-normally distributed continuous variables were reported as medians and interquartile ranges (IQR).

The chi-squared test was used for categorical variables, and the binomial test was applied for probability determination. For continuous variables, normality was assessed using the Shapiro-Wilk test, and homogeneity of variance was evaluated using Levene's test. Independent sample *t*-tests were applied if assumptions of normality and homogeneity of variance were met; otherwise, Wilcoxon rank sum tests were used. A *P*-value below 0.05 was considered statistically significant.

To explore the relationship between environmental temperature, seasonal changes, and ATR, we conducted analyses treating temperature groups and seasons as categorical variables. Correlation analyses were performed using a binomial test.

## Results

### Baseline participant characteristics

A total of 379 patients with ATR were identified from three provinces in China—Hubei, Shandong, and Inner Mongolia—spanning a 3-year period. Participants ranged in age from 19 to 75 years, with a mean age of 39.2 ± 9.7 years. Most cases were male (84.2%, *n* = 319), and sports-related injuries accounted for 86.3% of all cases (Table 1).

**Table 1:** Baseline characters of three multiple centers.

**Characteristic**	**Total**	**Hubei**	**Shandong**	**Inner Mongolia**
*n*	379	133	147	99
Age (years)	39.2 ± 9.7	38.1 ± 9.9	39.7 ± 10.6	40.0 ± 7.7
**Gender (%)**				
Male	84.2 (80.1, 87.7)	97.0 (92.5, 99.2)	91.8 (86.2, 95.7)	55.6 (45.6, 65.5)
Female	15.8 (12.3, 19.9)	3.0 (0.8, 7.5)	8.2 (4.3, 13.8)	44.4 (34.5, 54.4)
**BMI (%)**				
Normal	45.9 (40.9, 51.0)	48.1 (39.5, 56.7)	37.4 (29.5, 45.3)	55.6 (45.2, 65.5)
Overweight	48.0 (43.0, 53.1)	47.4 (38.8, 56.0)	51.0 (42.8, 59.2)	40.4 (30.6, 50.2)
Obese	6.1 (3.7, 8.5)	4.5 (0.9, 8.1)	11.6 (6.3, 16.8)	4.0 (0.1, 8.0)
**Blood pressure (mmHg)**				
Systolic pressure	130.6 ± 12.1	125.8 ± 10.2	134.5 ± 15.1	131.4 ± 5.4
Diastolic pressure	79.7 ± 8.4	79.7 ± 8.0	81.0 ± 10.3	77.6 ± 4.7
Blood glucose (mmol/L)	5.08 ± 0.73	5.02 ± 0.70	4.90 ± 0.78	5.40 ± 0.59
**Smoking status (%)**				
Yes	15.3 (11.8, 19.3)	15.0 (9.4, 22.3)	12.9 (8.0, 19.4)	19.2 (12.0, 28.3)
No	84.7 (80.7, 88.2)	85.0 (77.7, 90.6)	87.1 (80.6, 92.0)	80.8 (71.7, 88.0)
Environmental temperature (°C)	19.1 (11.0, 24.8)	22.2 (15.4, 28.5)	17.5 (10.9, 22.3)	18.5 (2.1, 23.7)
**Mechanism of rupture (%)**				
Sports related	86.3 (82.4, 89.6)	88.0 (81.2, 93.0)	82.3 (75.2, 88.1)	89.9 (82.2, 95.0)
Non-sports related	13.7 (10.4, 17.6)	12.0 (7.0, 18.8)	17.7 (11.9, 24.8)	10.1 (5.0, 17.8)

BMI, body mass index.

n, number; %, percentage; mean ± SD for continuous variables; % (95% confidence interval CI) for categorical variables.

The incidence of injuries varied significantly across age groups and BMI groups. Among all participants, the highest incidence was observed in individuals aged 30–39 years (*n* = 143), while the lowest incidence occurred in those under 20 years. The peak occurred in overweight (*n* = 178), with obese showing the fewest injuries (Table 2).

**Table 2:** Age and BMI distribution characteristics of three multiple centers.

**Characteristic**	**Total (*****n*** = **379)**		**Hubei (*****n*** = **133)**		**Shandong (*****n*** = **147)**		**Inner Mongolia (*****n*** = **99)**	
	***n*** **(proportion)**	***P*** **-value**	***n*** **(proportion)**	***P*** **-value**	***n*** **(proportion)**	***P*** **-value**	***n*** **(proportion)**	***P*** **-value**
**Age (years)**								
< 20	2 (0.0053)	< 0.001	1 (0.0075)	< 0.001	1 (0.0068)	< 0.001	0 (0)	/
20–29	54 (0.1425)	0.53	21 (0.1579)	0.35	21 (0.1429)	0.557	12 (0.1212)	0.33
30–39	143 (0.3773)	< 0.001	58 (0.4361)	< 0.001	54 (0.3673)	< 0.001	31 (0.3131)	< 0.001
40–49	129 (0.3404)	< 0.001	35 (0.2632)	< 0.001	50 (0.3401)	< 0.001	44 (0.4444)	< 0.001
50–59	40 (0.1055)	0.02	13 (0.0977)	0.08	15 (0.1020)	0.093	12 (0.1212)	0.33
60–69	8 (0.0211)	< 0.001	5 (0.0376)	< 0.001	3 (0.0204)	< 0.001	0 (0)	/
>69	3 (0.0079)	< 0.001	0 (0)	/	3 (0.0204)	< 0.001	0 (0)	/
**BMI**								
Normal	174 (0.4591)	< 0.001	64 (0.4812)	< 0.001	55 (0.3741)	0.166	55 (0.5556)	< 0.001
Overweight	178 (0.4802)	< 0.001	63 (0.4737)	0.001	75 (0.5102)	< 0.001	40 (0.4444)	0.083
Obese	27 (0.0607)	< 0.001	6 (0.0451)	< 0.001	17 (0.1156)	< 0.001	4 (0)	< 0.01

The proportions of age and BMI relative to the test values are 0.1429 and 0.3333, respectively.

BMI, body mass index; n, number.

### Association between temperature and ATR

**Figure 2:**
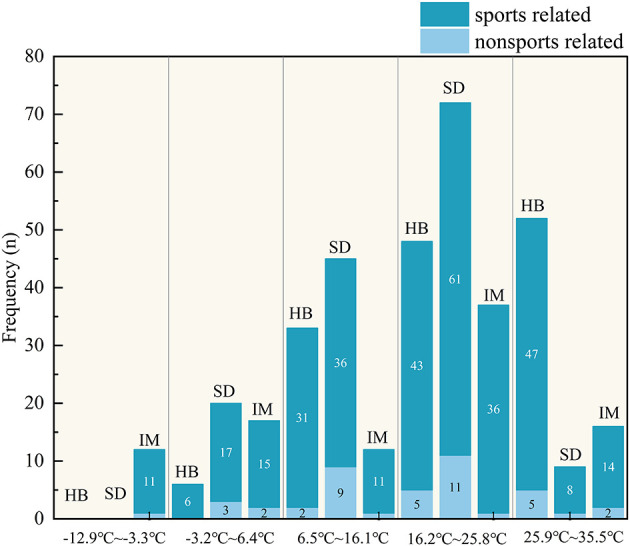
Temperature distribution of Achilles tendon rupture in sports related and non-sports related cases. HB, Hubei province; SD, Shandong province; IM, Inner Mongolia.

Analysis of environmental temperature revealed a significant association with the incidence of ATR. ATR incidence was highest at temperatures 16.2–25.8 °C, a pattern that was observed consistently across Shandong and Inner Mongolia Provinces (*P* < 0.001), However, the highest incidence occurs at temperatures 25.9–35.5 °C in Hubei. Conversely, the injury rate was lowest at temperatures ranging from −12.9 to −3.3 °C, a pattern observed in all three provinces of Hubei, Shandong, and Inner Mongolia (Figure 2). This trend persisted for sports-related injuries, all demonstrating robust statistical significance (all *P* < 0.005).

### Association between seasons and ATR

**Figure 3:**
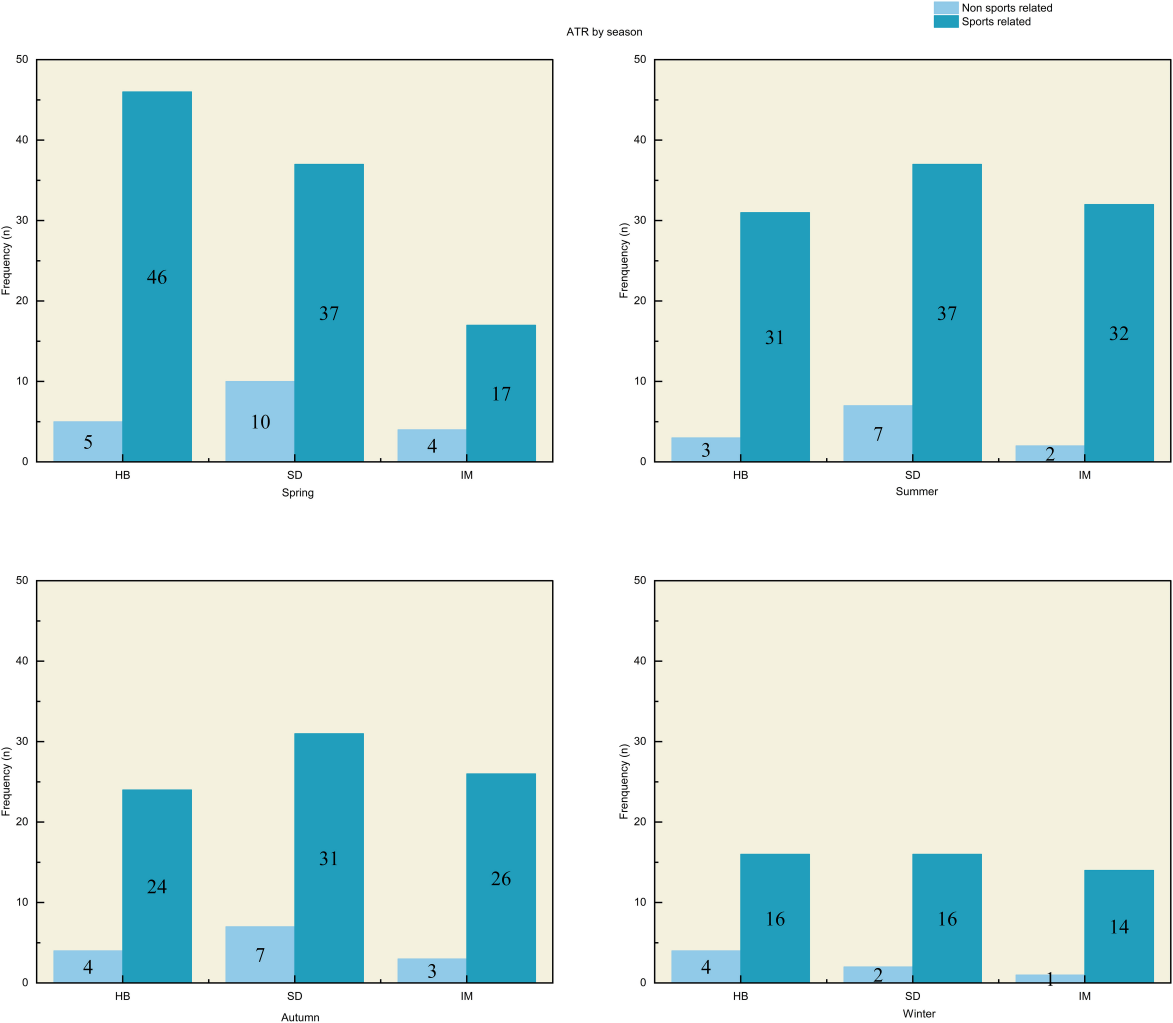
**(A–D)** The frequency of Achilles tendon ruptures in spring **(A)**, summer **(B)**, autumn **(C)**, winter **(D)**. HB, Hubei province; SD, Shandong province; IM, Inner Mongolia.

**Figure 4:**
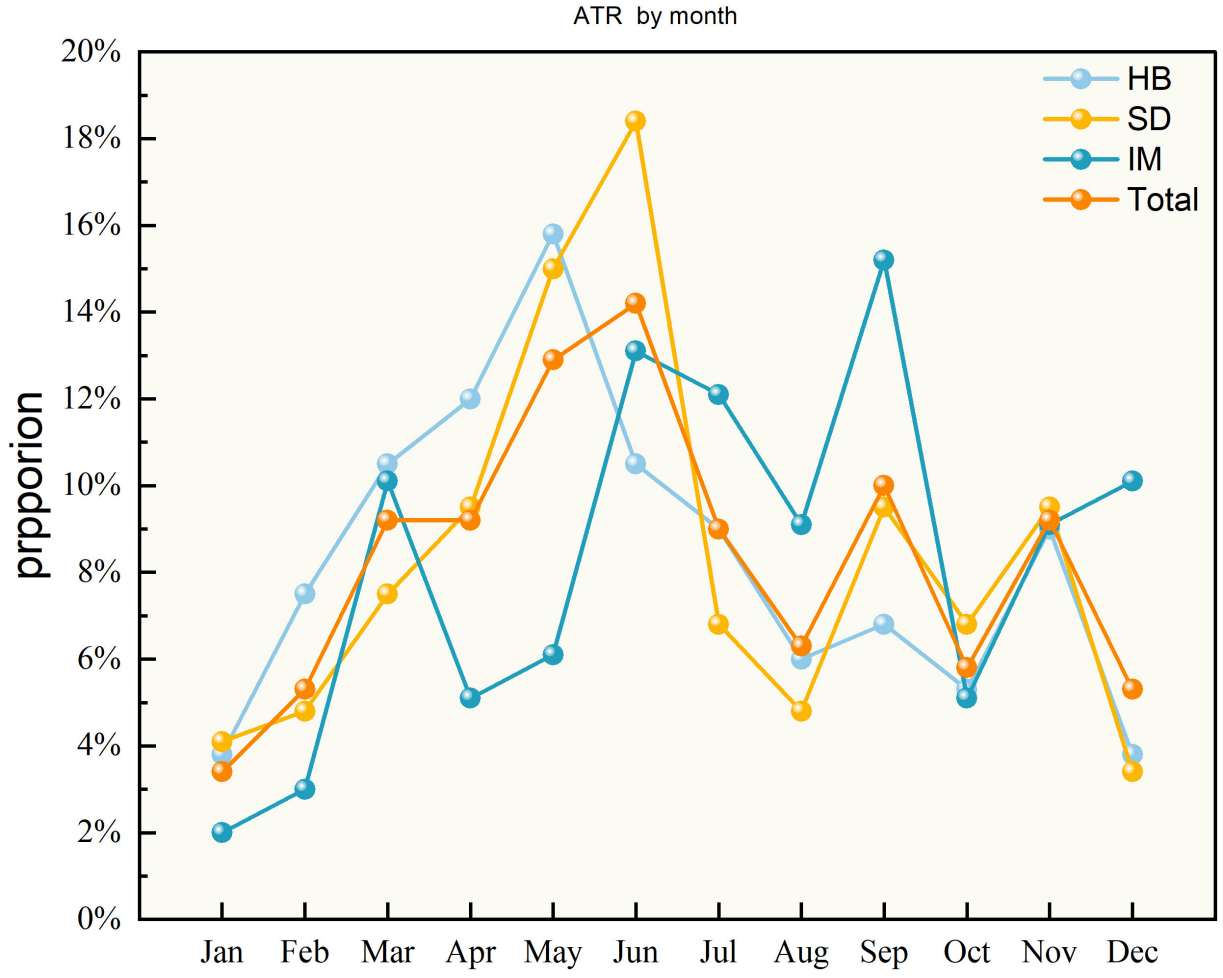
Achilles tendon rupture cases in different months. HB, Hubei province; SD, Shandong province; IM, Inner Mongolia.

Seasonal analysis revealed that the highest incidence of ATR occurred in spring (*n* = 119; *P* = 0.003), while winter exhibited the lowest incidence (*n* = 53; *P* < 0.001). No statistically significant differences in injury rates were observed for summer or autumn. This seasonal pattern was consistent for sports-related injuries, where spring showed the highest incidence, and winter the lowest. Regionally, we noted differences in the seasonal peak of injury occurrence. In Hubei and Shandong, the peak occurred in spring, in Inner Mongolia, it shifted to summer (Figure 3). When stratified by month, injury rates peaked in June followed by a decline during the summer and autumn months, with January showing the fewest injuries (Figure 4). Regional differences in the timing peak injuries were noted: in Hubei, the peak occurred in May (15.8%); in Shandong, it shifted to June (18.4%); and in Inner Mongolia, the peak was observed in September (15.2%).

**Figure 5:**
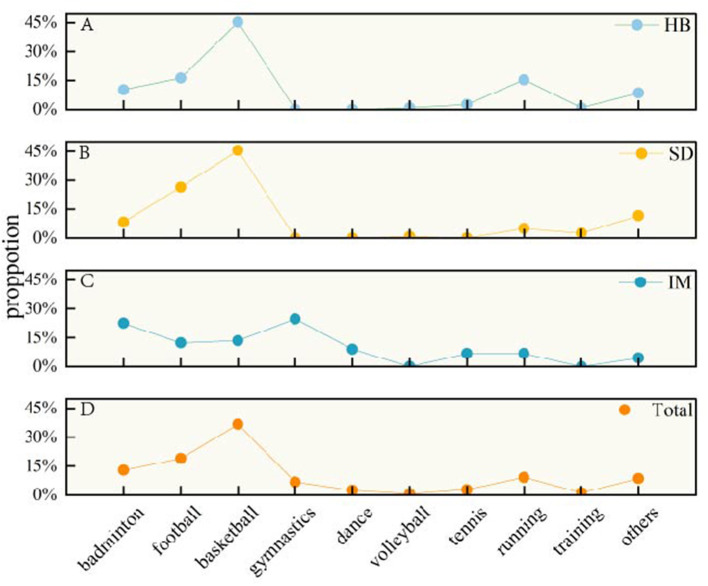
**(A–D)** Distribution of Achilles tendon rupture across various sports in Hubei **(A)**, Shandong **(B)**, Inner Mongolia **(C)**, and overall **(D)**. HB, Hubei province; SD, Shandong province; IM, Inner Mongolia.

**Figure 6:**
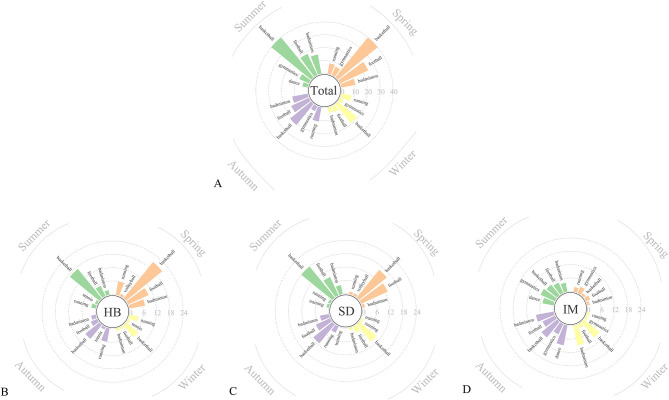
**(A–D)** Seasonal distributions of Achilles tendon rupture across various sports in Hubei **(A)**, Shandong **(B)**, Inner Mongolia **(C)**, and overall **(D)**. HB, Hubei province; SD, Shandong province; IM, Inner Mongolia.

For sports-related injuries, basketball was the most common activity associated with ATR (*n* = 120, 31.7%, *P* < 0.001), followed by football (*n* = 62, 16.4%, *P* = 0.001) and badminton (*n* = 44, 11.1%, *P* = 0.332; Figure 5). In Inner Mongolia, gymnastics and badminton were the primary sports contributing to ATR. Despite seasonal fluctuations, basketball demonstrated consistently high injury rates across all seasons, with the highest incidence in spring (*n*=40) and the lowest in winter (*n* = 19; Figure 6). However, no significant differences in injury rates were observed across different sports and seasons (*P* = 0.098). Additionally, no significant variation in injury rates was found when comparing temperature effects across different sports (*P* = 0.478).

## Discussion

This retrospective study identified a significant association between environmental temperatures at the time of injury, seasonal variations, and the incidence of ATR across multiple centers in China. Our analysis revealed that the incidence rate of ATR peaks when the temperature reached 16.2–25.8 °C, and significantly decreased when the temperature fell below 16.2 °C, reaching its lowest value at −12.9 to −3.3 °C. This pattern was general consistently observed across the three cities included in the study. However, in Hubei, the peak incidence of Achilles tendon rupture occurred at temperatures between 25.9 and 35.5 °C, which may be attributed to the influence of climatic conditions and regional characteristics. Similarly, the highest incidence of ATR occurred in spring, while winter exhibited the lowest rates. These findings suggest a possible association between ATR occurrence and environmental factors, such as temperature and seasonal variation.

To our knowledge, this study is the first to specifically examine the relationship between environmental temperatures at the time of injury and ATR. The observed trend of increased ATR rates at temperatures (16.2–25.8 °C) aligns with physiological mechanisms that may predispose tendons to rupture (25, 26). Cooler temperatures are associated with vasoconstriction, which reduces skin blood flow, oxygen delivery, and tendon vascular supply (27). Hypothermia further suppresses the expression of pro-angiogenic factors and reduces muscle vascular volume, effects that are critically relevant to the perfusion of the Achilles tendon—a structure inherently characterized by sparse vascularization (28, 29). In addition, impaired vascular endothelial function at temperatures ≤ 34 °C exacerbates this issue by reducing the expression of proangiogenic factors, weakening the tendon's resilience (30, 31). The temperature interval of 16–25 °C may represent a “critical range” for collagen fiber remodeling, potentially inducing instability in vascular regulation and compromising Achilles tendon blood supply. The hypovascular zone 2–6 cm proximal to the insertion (a known anatomical area of weakness) appears particularly susceptible to thermal variations. Lower temperatures may thus aggravate local ischemic degeneration and diminish the tissue's regenerative capacity in this vulnerable region.

Low temperatures also negatively affect tendon mechanical properties, reducing flexibility and neuromuscular function while increasing collagen stiffness (32–34). Under these conditions, type III collagen, which is less elastic and weaker, may replace type I collagen, with the disappearance of the normal arrangement of parallel collagen fibers, the tendon becomes cartilage-like and cannot withstand tensile force, resulting in increased stiffness and loss of viscoelasticity, further compromising tendon integrity and increasing rupture risk (35, 36). Although ambient temperatures within the range of 16–25 °C are generally considered to lie within the human thermoneutral comfort zone, inadequate warm-up under these conditions may result in a suboptimal functional state of the Achilles tendon. This can lead to insufficient viscoelasticity of collagen fibers, delayed collagen remodeling, and an increased susceptibility to cumulative microdamage during physical activity.

The seasonal variation in ATR incidence, with a peak in spring and a trough in winter, is consistent with evidence that physical activity is itself seasonally patterned. Longitudinal and device-based studies have shown that leisure-time and total physical activity levels are generally higher in spring and summer and lowest in winter (37, 38). Spring offers moderate temperatures and longer daylight, which favors outdoor sport and may increase cumulative tendon loading as competitive seasons conclude. By contrast, cold temperatures, shorter days and adverse weather in winter discourage outdoor activity and may contribute to the lower ATR rates observed in our cohort (39). In our data, ATR incidence was consistently lowest at very low temperatures and highest within mild-to-warm ranges across regions, suggesting that ambient temperature itself may contribute to rupture risk, superimposed on seasonal changes in activity volume. These observations indicate that seasonal differences in ATR likely reflect a combination of higher activity volume and environmental conditions, rather than either factor alone. However, because individual activity volume and training load were not available in our retrospective dataset, we could not formally separate the effects of physical activity from those of temperature. Accordingly, season and temperature in our analyses should be interpreted as complementary contextual markers that together capture behavioral and environmental influences on rupture risk, rather than fully independent causal determinants. Prospective studies incorporating device-based physical-activity monitoring and detailed training histories will be needed to disentangle these pathways more precisely.

Our regional analysis revealed a unique pattern in Inner Mongolia, where ATR incidence peaked in summer, in contrast to the spring peaks observed in Hubei and Shandong. This difference may be influenced by regional climate factors, such as sunshine duration, which directly affects physical activity levels. These variations emphasize the intricate interplay between environmental conditions, regional behaviors, and ATR risk. However, other studies have reported divergent patterns. For instance, ATR incidence were peaked in autumn in Denmark (40), and during winter and spring in Sweden (41), while no significant seasonal trends were observed in Scotland (42) or Canada (43). These discrepancies likely reflect the influence of latitude, climate, and cultural differences on seasonal activity patterns, further underscoring the multifactorial nature of ATR risk.

Our findings also highlight a significantly higher ATR incidence in those aged 30–39 years, while the lowest incidence occurred in those under 30 years, with sports-related injuries being the leading cause. This observation aligns with findings from Edmonton (43) and Seoul (20), which suggest that the mechanism of ATR differs in younger patients compared to older patients (42). The higher susceptibility in this demographic may be attributed to the prevalence of intermittent high-intensity physical activity, commonly referred to as “weekend warrior” behavior, which can compromise tendon function and elevate rupture risk. Older patients were more likely to sustain complete ATR, potentially due to age-related degenerative changes in tendon structure or the surgical focus of our cohort. Additionally, patients who are overweight exhibit a higher incidence of ATR. This may be due to the sustained excessive mechanical stress induced by obesity leads to a progressive accumulation of microtrauma within the Achilles tendon, ultimately contributing to tendon degeneration through mechanobiological pathways.

Basketball emerged as the most common sport associated with ATR, reflecting its popularity and the physical demands of rapid movements, jumping, and sprinting. While sports such as racquet sports and football also contribute to ATR risk, their impact appears to vary by region and population. For example, studies from Denmark and Hungary reported no significant correlation between ATR and environmental temperature for these activities (26, 44, 45). In our cohort, however, the activity setting (indoor vs. outdoor) at the time of injury was not systematically documented in the medical records. This is particularly relevant for basketball, which can be played both indoors and outdoors. As a result, some events may have occurred in temperature-controlled indoor environments, potentially attenuating the observed association between outdoor temperature and sports-related ATR. Nevertheless, the seasonal and temperature patterns we observed are likely to reflect broader environmental influences on participation in high-load sports rather than the exact microclimate experienced by each individual. These findings highlight the need for targeted interventions, such as regular exercise and proper warm-up routines, to maintain tendon health and minimize injury risk, especially among amateur athletes or individuals with sedentary lifestyles (46, 47).

## Strengths and limitations

This study has several limitations. First, all data were sourced from patients who underwent surgery in three hospitals, which may restrict the generalizability of our findings to the broader population within these regions. Non-surgical cases of ATR were excluded, potentially introducing selection bias and underestimating the true incidence of ATR. Second, as a retrospective study, we relied on pre-existing medical records, which may have been incomplete or imprecise, contributing to potential biases in data collection and analysis. Third, this study was not designed as an epidemiological investigation and did not estimate the population-level incidence of ATR. While our sample size was adequate for exploring associations, it was smaller than that of some comparable studies, potentially limiting the statistical power of our findings.

Additionally, while ATR is influenced by numerous intrinsic and extrinsic factors, our analysis was limited to a restricted set of variables, such as gender, age, height, BMI, smoking history, blood glucose levels, and type of exercise. Other potentially important factors, including physical activity intensity, training habits, competitive vs. recreational athletic level, footwear type, training surface and conditions, and biomechanical characteristics, were not assessed because they were not systematically documented in the medical records. This study represents a retrospective analysis of a case series without a control group; therefore, it was not possible to assess the independent impact of covariates such as gender or smoking status on ATR risk. Furthermore, environmental factors such as humidity or wind speed, which may interact with temperature to influence ATR risk, were not available and were therefore excluded from our analysis. Future studies incorporating these variables and broader populations could provide a more comprehensive understanding of the complex interactions influencing ATR risk.

## Conclusions

This study demonstrates a significant association between environmental temperature, seasonal variations, and ATR incidence in three provinces of China. ATR rates were highest at temperatures 16.2–25.8 °C and peaked in spring, emphasizing the role of environmental factors. This work is the first to validate that relatively lower outdoor temperatures are a new risk factor for Achilles tendon rupture. The more intense physical activities associated with warmer weather may provide a plausible explanation. Awareness of risk factors and proper warm-up education should contribute to the prevention of Achilles tendon ruptures on a broader basis. Regional differences highlight the need for tailored preventive strategies. Future research with larger cohorts is essential to validate these findings and improve preventive measures.

## Data Availability

The original contributions presented in the study are included in the article/supplementary material, further inquiries can be directed to the corresponding author.
